# An Appeal to Supernatural Force

**Published:** 2010

**Authors:** Rachel Hajar

**Affiliations:** *Submitted by Rachel Hajar, M.D*. Hamad Medical Corporation, Doha, Qatar Extracted from Lyons, AS, II Petrucelli A. 1987. Medicine: An Illustrated History. New York: Harry N. Abrams, Inc

**Figure:**
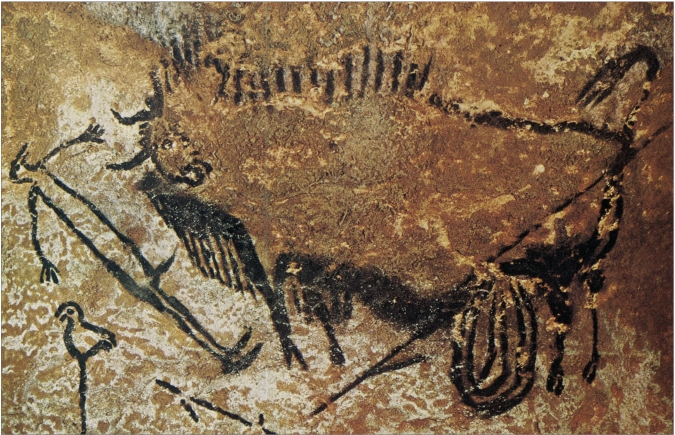
Pre-Historic Painting (c.15,000-10,000 B.C.) Lascaux Cave, France A wounded bison, intestines spilling out stands over an apparently dead human figure. The scene has been interpreted as an appeal to supernatural forces rather than as a simple record of a hunting incident

## MEDICINE IN PREHISTORY

### What diseases plagued early humans?

Before there were humans on earth, there was disease. Investigations of human remnants from historic periods have uncovered many disease entities, for instance, tuberculosis and parasitic infestation in the mummies of ancient Egypt. Paleopathologists search for possible clues among the surviving prehistoric skeletons and artifacts. Fractures seem to have been common, and while some healed with little deformity, others show effects of infection (osteomyelitis), poor apposition of the bony fragments, and extensive calluses (bone “scars” associated with healing). Arthritis in dinosaurs and prehistoric bears was evidently so common that scholars have named it “cave gout”. Cavities were a problem in Paleolithic and Neolithic times. They became a common disorder in ancient Egypt, especially in its later history. There is no clear-cut evidence of prehistoric diseases of the soft parts for obvious reasons – unlike bones, tissues do not last. No bodies or organs earlier than 4000 BC have been discovered. Microscopic imprints on rocks seem to indicate the presence of bacteria in prehistoric periods, but we have no way of knowing whether these were pathogenic.

In the mummies of early Egypt, arteriosclerosis, pneumonia, urinary infections, stones, and parasites have been identified, which may suggest that such conditions also prevailed in earlier unrecorded periods. We do not know whether prehistoric man suffered arteriosclerosis, but its very presence – sometimes in advanced degree – in ancient Egyptian mummies may have a bearing on our modern ideas concerning its causes. If early humans existed without strains similar to those of technically advanced civilization, then stress would have an unlikely relation to arteriosclerosis. It appears, however, that man’s illnesses, for the most part have been continuations of the diseases and bodily mechanisms of the creatures which preceded or accompanied him.

### Prehistoric man’s lifespan

Bones from Paleolithic, Mesolithic, and Neolithic periods strongly suggest that a lifetime averaged approximately 30 or 40 years. In virtually all reported studies, men seemed to have lived longer than women, the common assumption being that pregnancy and childbirth were responsible for the difference. Evidence suggests that even after childbearing age, women had shorter life expectancies than men of comparable age (the opposite of present experience). Possibly, chronic malnutrition, starting in infancy and continuing through childhood, made women less resistant to illness. According to this theory, men and boys, as leaders, hunters, and warriors, were considerably better fed than women and girls who were the home laborers, crop cultivators, and childbearers.

### How did early humans treat their illnesses?

Some writers have surmised from the self-treatment of sick animals – licking wounds, delousing one another, and eating emetic grasses – that prehistoric man also employed similar care. We do not know whether any treatment was used by the earliest humans. Good outcome of sickness or injury does not necessarily mean that therapy was employed; many illnesses and wounds heal themselves. In one collection of prehistoric specimens, over half of the fractured bones seem to have healed with good results, but well-aligned healing of fractured bones of wild animals has also been observed. Furthermore, we have to guess at the knowledge of the body possessed by early humans. Cave pictures have received considerable attention and a variety of interpretations.

Did prehistoric people develop a cult of healing? A painting in the Trois Freres cave in France of an erect, possibly dancing figure with deer head or mask has been thought by some to represent the first shaman, or healing priest. Another Paleolithic fragment shows a reindeer stepping over a supine pregnant woman. Was this ritual to transmit strength or was it a medical method to hasten labor?

In the Neolithic period (about 10,000–7000 BC), humans apparently shifted from food gathering to food producing. One can assume that medicinal herbs were among the plants grown, but whether and when they were recognized to possess healing properties is not known. It is also possible that more secure shelter and more regularly available food led to fewer illnesses. With the use of tools, Neolithic men and women became craftsmen. They might also have used implements for surgical purposes since examples of trepanation dating to the Neolithic period have been discovered in France. Signs that the skull wound was healing indicate that a fair proportion survived the operation. Trepanation might have had a magico-medical purpose of letting out a demon, as has been observed in some primitive people. On the other hand, it could have been a treatment for fractures or a means of removing bone splinters. Indeed, trepanation might have been employed at different times for all of the above reasons.

Although considerable knowledge of prehistory is gained from fossils, paleontology, physical anthropology, paleopathology, sculpture, and cave art, the answers for many of our questions are still conjectural. Folklore, known medical practices of primitive people, and the archaeological and literary evidences of ancient civilizations may well give additional indications of what preceded them, but this information can also be misleading since primitive societies and ancient cultures themselves have often undergone change through the centuries.

